# Changes in NK Cell Subsets and Receptor Expressions in HIV-1 Infected Chronic Patients and HIV Controllers

**DOI:** 10.3389/fimmu.2021.792775

**Published:** 2021-12-16

**Authors:** Zhi Zhang, Ying Zhou, Jing Lu, Yuan-Fang Chen, Hai-Yang Hu, Xiao-Qin Xu, Geng-Feng Fu

**Affiliations:** Department of HIV/STD Control and Prevention, Jiangsu Provincial Center for Disease Control and Prevention, Nanjing, China

**Keywords:** NK cell, NK cell subset, receptors of NK cell, HIV controller, plasma cytokine

## Abstract

Natural killer (NK) cells are major effectors of the innate immune response and purported to play an influential role in the spontaneous control of HIV infection. In the present study, we compared the phenotypes of NK cells in the peripheral blood of three groups of subjects with chronic HIV-1 infection, HIV controllers, and healthy donors. The results showed that CD56^+^/CD16^-^ NK cell subsets decreased in chronic patients and remained unchanged in controllers. Notably, we found that people living with chronic HIV-1 infection had suppressed NKp80, NKp46, and NKG2D expressions on NK cells compared to healthy donors, while HIV controllers remained unchanged. In contrast, NKG2D expression was substantially higher in controllers than in chronic patients (M=97.67, p<0.001). There were no significant differences in inhibitory receptors KIR3DL1 and KIR2DL1 expressions. In addition, plasma cytokine IFN-γ, TNF-α and IL-12showed higher levels in HIV controllers compared to chronic patients. Overall, our study revealed that, as compared to chronic patients, HIV controllers show an increased activating receptors expression and higher number ofCD56^+^/CD16^-^NK cell subset, with increased expression levels of plasma cytokines, suggesting that higher immune activation in controllers may have a key role in killing and suppressing HIV.

## Introduction

People infected with HIV-1 virus experience a short acute HIV infection period and then enter an asymptomatic HIV-infected stage. The disease will progress to AIDS in the majority of the patients. However, a small proportion (less than 1%) of infected individuals, referred to as ‘HIV controllers’ (1), can maintain continuous control over viral replication even without antiretroviral therapy (1–3). Generally, there are two categories of controllers: elite controllers and viremic controllers. While the viral load is undetectable with conventional assays in the elite controllers, viremic controllers decrease and present with low but detectable virus levels (less than 2000 copies RNA/mL) (2, 4).

Although the controllers cannot eradicate HIV-1, their immune systems exhibit extraordinary ability to control viral replication. The mechanism underlying this antiviral immunity remains unclear. Some data suggest that NK cells mediated response may contribute to the sustained viral control alongside traditional adaptive immune responses in some controllers (2, 5, 6). Natural killer (NK) cells, which account for up to 15% of peripheral blood lymphocytes, are major effectors of the innate immune response (7, 8). Recent data showed that NK cells might play an influential role in resistance against HIV (9, 10) and serve to control HIV infection in long-term non-progressors (LTNPs) (11, 12).

Based on the expression of CD16 and CD56, NK cells can be grouped into three or five subsets (8, 9, 11). Many data suggest disproportionality of NK cell subsets exists in HIV-1 infected people (13, 14). The imbalance of subset may impair the function of NK cells (15). Furthermore, NK cell receptors also play a vital role in NK cell responses. NK cells possess either activating or inhibitory receptors and bind to their cognate ligands on the surface of target cells (16). Cytotoxicity of NK cells is regulated by the balance with the expressions of these receptors (17). Inhibitory receptors can effectively prevent NK cells from attacking normal cells, thus becoming the principal determinant of NK cells’ self-tolerance (18). In humans, KIR3DL1 and KIR2DL1 were two of the most prominent NK cell receptors (19). However, potential targets with downregulated class I expressions (like transformed or infected cells) may stimulate activator receptors to release signals and instigate NK cell cytolytic activities (18). In concert with the loss of inhibitory signals, activation signals *via* NK receptors such as NKp30, NKp44, NKp46, NKG2D, and NKp80 mediate the activation of NK cells. Some studies showed that persons living with viremic HIV-1 have altered expression and function of several important activating and inhibitory NK cell receptors (20–23). Furthermore, some researches showed that the cytokines also varied in HIV-1 infected individuals (24–26).

To date, in both anti-HIV prevention and therapy, the majority of approaches focus on manipulating human immune responses to reduce or eliminate these persisting reservoirs by neutralizing antibodies. However, knowledge about the innate immune components that may lead to viral reservoir reduction is limited (27). This study aimed to assess the expression of subsets and receptors of NK cells and determine the underlying mechanism of disease progression in HIV controllers.

## Materials and Methods

### Study Population

This study screened the untreated HIV infected patients in Jiangsu Province who had an absolute count of CD4+ T cell greater than 500 and had been infected for five years or more since diagnosis from 2014 to 2019. The viral load, immunophenotype and plasma cytokines were tested. 65 individuals volunteered to participate in this study. The patients were classified into two groups according to the level of HIV viral load: HIV controllers (n = 9, viral loads ≤ 2,000 copies/mL), people with chronic HIV-1 infection, hereafter referred to as chronic patients (n = 56, viral loads > 2,000 copies/mL). Control samples were HIV negative obtained from a group of 120 healthy adults having a physical examination (hereafter referred to as healthy donors). Descriptive characteristics of the study participants are presented in **Table 1**.

**Table 1:** Characteristics of HIV-1 infected Patients and Healthy Donors in Jiangsu Province during 2014 to 2019.

	HIV Controller	Chronic patient	Healthy Donor
N	9	56	120
Gender	F:5	F:15	F:60
	M:4	M:41	M:60
Age(Y)	M:41.11	M:39.61	M:42.79
	SD:9.29	SD:10.08	SD:14.10
	R:26-57	R:7-56	R:17-80
Mean years since HIV diagnosis	M:7.33	M:7.13	N/A
	SD:1.66	SD:1.51	
	R:6-10	R:6-12	
Viral load Copies/mL	M:820.83	M:62657.18	N/A
	SD:375.69	SD:104669	
	R: NDT-1267	R:2544-516113	

‘NDT’, ‘Not Detected’, means lower than the detection limits; F, female; M, male; M, mean; R, range; SD, standard deviation; N/A, not applicable.

Peripheral blood was collected in EDTA anticoagulated vacuum tubes from ten cities of Jiangsu province including Nanjing, Xuzhou, Wuxi, Suzhou, Changzhou, Nantong, Lianyungang, Yancheng, Yangzhou, Zhenjiang and Taizhou, then transported to the Jiangsu Provincial Center for Disease Prevention and Control (Jiangsu CDC) for testing. The blood samples were analyzed using flow cytometry (BD FACS Calibur and Aria II) and HIV-1 viral load measurement.

### Counts of the CD4 T-Cells and NK Cells

We tested anticoagulated whole blood collected from HIV-1 infected individuals and healthy donors within 24 hours for CD4 and NK cell counts. The 50-µL whole blood sample was stained with mixed antibodies (CD4 T cell: FITC-CD3, PE-CD8, = PerCP-CD45 APC-CD4; NK cell: FITC-CD3 PerCP-CD45 = PE-CD56+CD16 APC-CD19) in Trucount Absolute Count Tubes (contained True count beads) and incubated for 15 min in the dark. Then we added 450 mL Erythrocyte Lysing Solution and stayed for 15 min. The labeled cells were analyzed by BD FACS Calibur flow cytometry, and 10^5^ cells were acquired.

### Phenotypic Analysis of NK Cells

**Figure 1:**
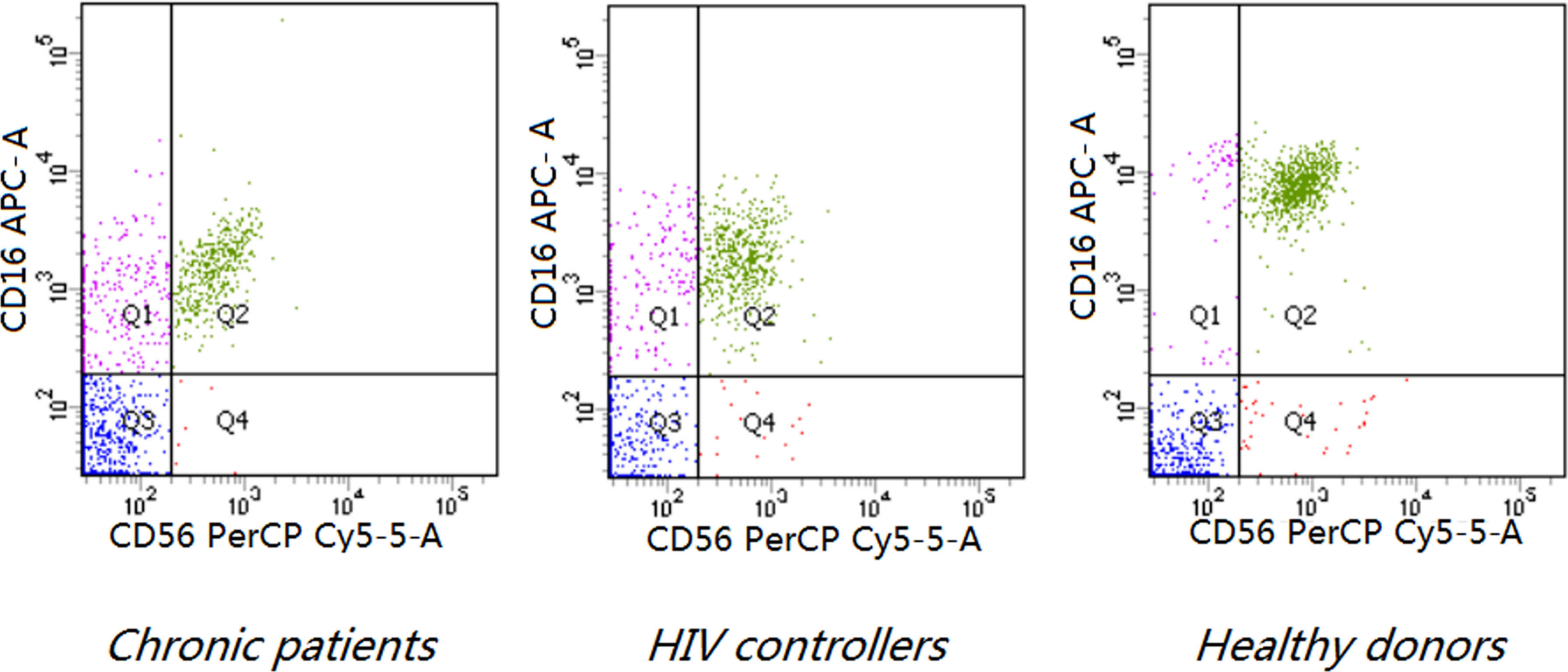
Phenotypic analysis of NK cells based on CD16 and CD56 expression and representative scatter plots of flow cytometric analysis of CD56 and CD16 staining on the CD3- lymphocyte of PBMCs of indicated donors (P < 0.0001). Both HIV controllers (7.92 ± 5.94,8.33 ± 5.82) and chronic patients (7.70 ± 4.21, 6.74 ± 5.32) had higher levels of CD3-CD16+CD56- subset and lower levels of CD3-CD16+CD56+ subset than healthy donors (4.51 ± 4.64, 15.00 ± 7.20) (P < 0.0001). The frequency of CD3-CD16-CD56+ subset decreased in chronic patients (0.54 ± 0.42) compared to healthy donors (0.97 ± 1.13) (P=0.0193).

All the cases were directly stained. The peripheral blood mononuclear cells (PBMCs) were incubated with the appropriate mAbs, anti-human CD3 Alex Flour 488/CD16 APC/CD56 PE-Cy5.5, and some natural killer cell receptors (the antibodies were -NKp80-PE, KIR2DL1-PE, KIR3DL1-PE, -NKp44-PE, -NKp46-PE, -NKp30-PE, -NKG2D-PE and Isotype Control Mouse IgG1,κ-PE) for 15 min. Lysed (Becton Dickinson) for 15 min and then washed by phosphate-buffered solution (PBS) 3 times. Four-color cytofluorometric analyses (Aria II; Beckton Dickinson) were used in analyzing the samples. Data were analyzed using Diva software (Beckton Dickinson) **(** **Figure 1** **)**. We further analyzed the phenotypes of the NK cells in a pulse diagram and counted 10,000 events of lymphocytes for each analysis. According to Isotype Control, the expression of receptors refers to NK cells that were gated.

### Level of HIV-1 Viral Load

The plasma of individuals infected by HIV-1 was determined using the Viral-load apparatus (Abbott M2000). The detection level in plasma was defined as 150 HIV-1 RNA copies/mL according to the sample volume of 200 µL.

### Statistical Analysis

The comparisons between counts of CD4+ T cell and NK cell and the percentage of NK cell subsets and NK cell phenotypesin HIV controllers, chronic patients, and healthy donors were done by variance analysis and least significant difference (LSD) test. Pearson correlation analysis was performed to assess correlation between CD4+ T cell and NK cell counts and correlation between NK cell receptors. The differences of plasma cytokine expression level between HIV controllers and chronic HIV-1 infected individuals were compared by Kruskal-Wallis test. The P values less than 0.05 were considered as significant.

### Data Availability

Data from this manuscript is available upon request from the authors.

## Results

### CD4^+^ T Cell Number Is Significantly Decreased in Chronic HIV-1-Infected Patients

As described in methods, HIV-1 infected individuals were categorized into two groups based on the level of HIV-1 viral load at a standard of 2000 copies/mL.9people were HIV controllers, and the other56 were chronic HIV-1 infected individuals.

The number of CD4^+^ T cells notably decreased in chronic HIV-1-infected patients (425.57 ± 131.98) compared to healthy donors (823.98 ± 313.92) and HIV controllers (762.67 ± 382.37) (*P*<0.0001). The mean CD4^+^ T cell count was slightly lower in the HIV controllers compared to healthy donors, but the difference was not statistically significant **(** **Table 2** **)**. The numbers of NK cells were comparable among the three groups (**Table 2**).

**Table 2:** The CD4^+^ T cell and NK cell Counts in HIV controllers, chronic patients, and healthy donors in Jiangsu Province during 2014 to 2019.

	HIV Controller (n = 9)	Chronic patient (n = 56)	Healthy Donor (n = 120)
CD4^+^ cell counts (cells/µL)	762.67 ± 382.37^a^	425.57 ± 131.98^ac^	823.98 ± 313.92
NK cell counts (cells/µL)	593.22 ± 589.90	425.07 ± 460.76	465.76 ± 290.37

‘a’ indicates a significant difference between HIV controller and chronic patient.

’b’ indicates a significant difference between HIV controller and healthy donor.

’c’ indicates a significant difference between chronic patient and healthy donor.

### NK Cell Subsets and Expression of NK Cell Receptors

Both HIV controllers (7.92 ± 5.94) and chronic patients(7.70 ± 4.21) had higher levels of CD3^-^CD16^+^CD56^-^ subset than healthy donors (4.51 ± 4.64) (*P*<0.0001). On the other hand, the CD3^-^CD16^+^CD56^+^ subset decreased in both chronic patients (6.74 ± 5.32) and HIV controllers (8.33 ± 5.82) (*P*< 0.0001).The frequency of CD3^-^CD16^-^CD56^+^ subset decreased only in chronic patients (0.54 ± 0.42) compared to healthy donors (0.97 ± 1.13) (*P*=0.0193). (**Table 3** and **Figure 1**).

**Table 3:** The Percentage of NK cell Subsets and NK Cell Phenotypes in HIV Controllers, Chronic Patients, and Healthy Donors in Jiangsu Provinceduring 2014 to 2019.

	HIV Controller (n = 9)	Chronic patient (n = 56)	Healthy Donor (n = 120)	Pvalue
CD3^-^/CD16^+^/CD56^-^	7.92 ± 5.94^b^	7.70 ± 4.21^c^	4.51 ± 4.64	<0.0001
CD3^-^/CD16^+^/CD56^+^	8.33 ± 5.82^b^	6.74 ± 5.32^c^	15.00 ± 7.20	<0.0001
CD3^-^/CD16^-^/CD56^+^	0.76 ± 0.53	0.54 ± 0.42^c^	0.97 ± 1.13	0.0193
CD3^-^/CD16^+^/CD56^+^/NKP80^+^	50.73 ± 20.95	47.00 ± 23.54^c^	57.08 ± 15.67	0.0038
CD3^-^/CD16^+^/CD56^+^/NKP46^+^	66.93 ± 18.34	62.31 ± 25.00^c^	73.88 ± 19.78	0.004
CD3^-^/CD16^+^/CD56^+^/NKP44^+^	3.24 ± 2.69	5.01 ± 7.67	5.62 ± 7.96	0.6287
CD3^-^/CD16^+^/CD56^+^/NKP30^+^	60.30 ± 19.68	59.56 ± 22.59	65.92 ± 19.78	0.146
CD3^-^/CD16^+^/CD56^+^/NKG2D^+^	97.67 ± 2.20^a^	89.66 ± 13.75^ac^	97.84 ± 5.56	<0.0001
CD3^-^/CD16^+^/CD56^+^/KIR2DL1^+^	56.39 ± 20.77	41.47 ± 24.22	46.68 ± 20.29	0.1025
CD3^-^/CD16^+^/CD56^+^/KIR3DL1^+^	19.72 ± 11.41	19.91 ± 16.85	22.52 ± 14.94	0.5405

‘a’ indicates a significant difference between HIV controller and chronic patient.

’b’ indicates a significant difference between HIV controller and healthy donor.

’c’ indicates a significant difference between chronic patient and healthy donor.

Next, we further analyzed the expression of functional receptors on the CD3^-^CD16^+^CD56^+^ since this subset was involved in the long-term control of viral replication. We examined five activating receptors (NKp80, NKp30, NKp44, NKp46, and NKG2D) and two inhibitory receptors KIR3DL1 and KIR2DL1. NKp80, NKp46, and NKG2D expression decreased in persons living with chronic HIV-1 infected than healthy donors (*P*<0.05).

**Figure 2:**
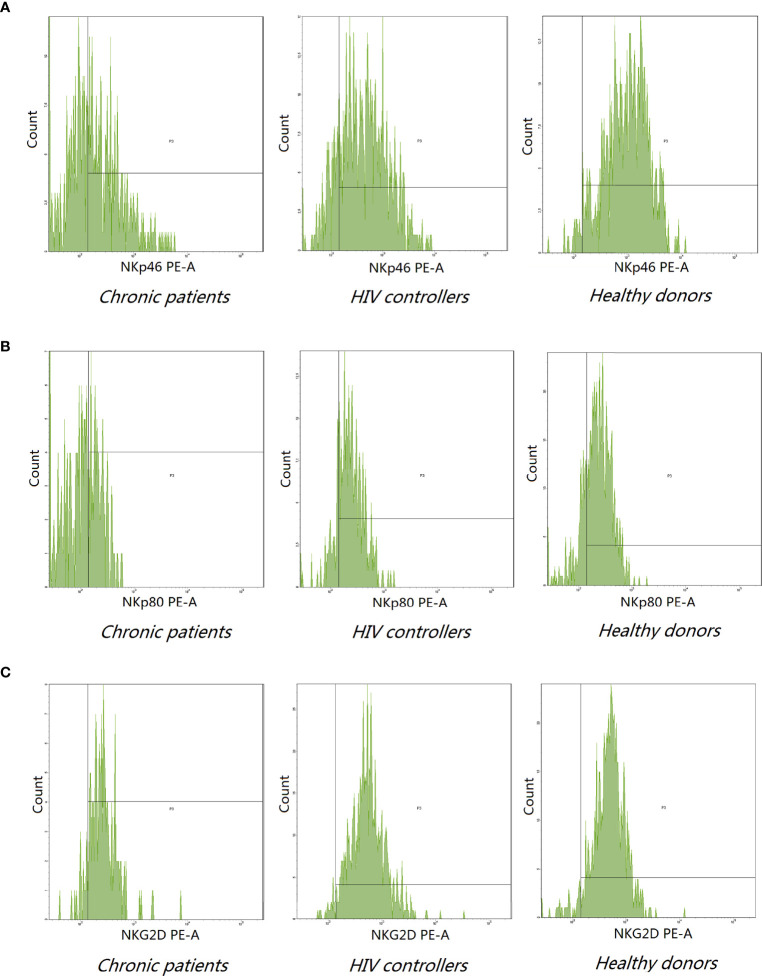
Representative histogram analysis of NKp46 **(A)**, NKp80 **(B)**, and NKG2D **(C)** on NK cells from the peripheral blood of indicated donors. **(A, B)** The expression of NKp46 (62.31 ± 25.00) and NKp80 (47.00 ± 23.54) decreased in chronic patients compared to healthy donors (NKp46:73.88 ± 19,P=0.004; NKp80:57.08 ± 15.67, P = 0.0038). **(C)** Chronic patients expressed lower level of NKG2D receptor (89.66 ± 13.75) compared to HIV controllers (97.67 ± 2.20) and healthy donors (97.84 ± 5.56) (P < 0.0001).

The expression of the NKG2D receptor significantly decreased in chronic groups compared with HIV controllers (*P*<0.05). Expression of the KIR3DL1 and KIR2DL1 inhibitory receptors and NKp44 and NKp30 activating receptors had no significant differences among the three groups (*P*> 0.05) (**Table 3** and **Figure 2**).

### Relationship of the Fact in HIV Controller Group

**Figure 3:**
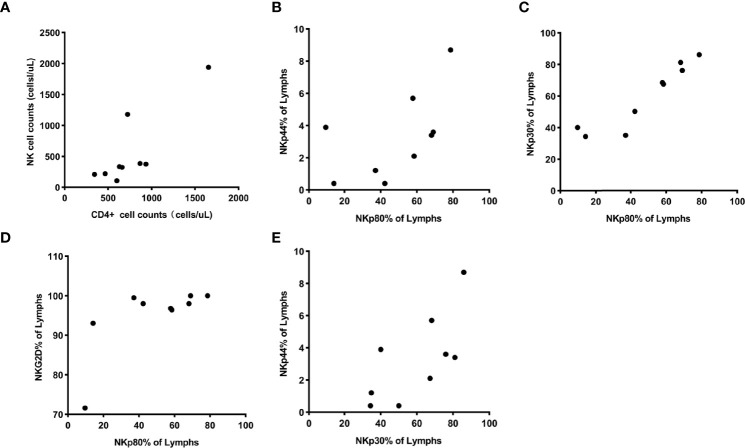
**(A)** showed the counts of CD4^+^ T cells were positively associated with counts of NK cells, and their correlation coefficient was 0.81 (P = 0.0084). **(B–D)** showed the expression of receptor NKp80 were positively associated with the expression of receptor NKp44, NKp30, and NKG2D, and the correlation coefficients were 0.70 (Pp = 0.0349), 0.96 (P < 0.0001), and 0.68 (P = 0.0476). Also, it **(E)** showed a positive association between the expression of NKp30 and NKp44 with a correlation coefficient of 0.73 (P = 0.0264).

In the HIV controller group, the counts of CD4^+^T cells positively correlated with the counts of NK cells (*P*< 0.05). NKp80 expression was positively associated with NKp44, NKp30, and NKG2D. NKp30 expression was positively associated withNKp44 (*P*< 0.05) (**Figure 3**).

### Plasma Cytokine of HIV Controllers and Chronic HIV-1 Infected Individuals

**Figure 4:**
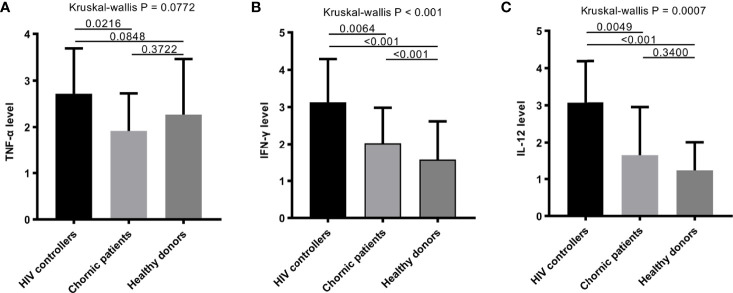
The plasma cytokines among HIV controllers, chronic patients and healthy donors. **(A–C)** Comparison of TNF-α **(A)**, IFN-γ **(B)**, and IL-12 **(C)** among HIV controllers, chronic patients and healthy donors. Some plasma cytokines, such as IFN-γ **(B)**, and IL-12 **(C)** in people with HIV infection performed higher than healthy donors. The level of TNF-α, IFN-γ and IL-12 in HIV controller group were significantly higher than the chronic group (P = 0.0064, P = 0.0216, P = 0.0049).

The levels of some plasma cytokines, such as IFN-γ, TNF-α and IL-12 in the HIV controller group were significantly higher than the chronic patients’ (**Figure 4**).

## Discussion

NK cells play a vital role in the immune response against HIV infection. The distribution of NK cell subsets and the expression of NK cell receptors had changed after HIV infection, which may play an essential part in helping HIV controllers kill and suppress HIV. The CD56^+^/CD16^-^, a subset of NK cells normally expressed in HIV controllers but decreased in chronic patients. Some activating receptors of NK cells in HIV controllers expressed almost the same as healthy donors, such as NKp80, NKp46, and NKG2D. In addition, HIV controllers expressed higher level of plasma cytokines of IFN-γ, TNF-α and IL-12.

NK cell subsets distribution had changed in HIV infectors(both in HIV controllers and chronic patients), except the expression of CD56^+^/CD16^-^ subset in HIV controllers. Our result was consistent with Mavilio’s study findings (28).The CD56^+^/CD16^-^ are a subset of NK cells that can perform high secretion of cytokines, such as IFN-γ and TNF-α, and regulate local immune response (29). IFN-γ, mainly produced by activated T cells and NK cells, can activate effector cell and enhance the activity of natural killer cells and the expression of tumor necrosis factors, such as TNF-α. This subset decreased in chronic patients that may reflect the cytokine secretion functional decline of NK cells, which performed normally in HIV controllers. In our study, the plasma cytokine and CD4 T-cell absolute counts results supported this conclusion. Compared with the chronic group, HIV controllers showed higher level of IFN-γ, TNF-α, IL12 and CD4 T-cells. IL-12, named NK cells stimulating factor (NSF), mainly acts on T cells and NK cells (30–32). IL-12 can induce NK cells to secrete IFN-γ to provoke antigen-presenting cells and promote Th1 differentiation. IL-12 activates the signal transduction, and transcriptional activator STAT4 promotes NK cells maturation and enhances the cytotoxicity of NK cells through IFN-γ production (33). That forms a cycle-promoting process. The plasma cytokine results also support this conclusion. The majority of NK cells are CD56^+^CD16^+^ subsets, which can release a high level of perforin and granzyme to kill target cells or tumor cells after activation, and have strong cytotoxicity (34). NK cells mediate antibody-dependent cell-mediated cytotoxicity(ADCC) and control their activation status through the accumulated signals received through activating and inhibitory receptors. CD16 can enhance this specific ADCC effect (10). The activating receptors include NKG2D, 2B4, NKp80, and natural cytotoxicity receptors (NCRs) NKp30, NKp44, and NKp46 (19). NCRs are primarily responsible for triggering NK-mediated lysis and are expressed almost exclusively on NK cells (35). Their expression on NK cells positively correlates with the degree of cytotoxic function, such as NKp44 only by activated NK cells, NKp46 is expressed by resting NK cells, and NKp30 by all NK cells (19). A variety of co-receptors that help to induce activation of NK-mediated cytolysis (36) have been identified (36). NKp80 is a C-type lectin-like homodimeric triggering co-receptor that stimulates cell cytotoxicity, which is expressed on NK cells alone (37). NK cell activation through NTB-A, 2B4, and NKp80 depends on the simultaneous triggering of other receptors such as NKp46 (19). Recent work suggested that synergistic interactions between activating receptors promote the induction of cytotoxicity in resting NK cells (38). The positive correlation between some activating receptors in our result was consistent with this research (38). Some data showed that NKp30 cooperates with NKp46 and NKp44 in NK cell-mediated cytotoxicity induction against most target cells (39). Some studies showed the expression of NCRs (NKp46, NKp30, and NKp44) remarkably decreased among viremic individuals along with a concomitant decrease in NK cytolytic activity (20, 22). The suppression expression of some activating receptors (NKp80, NKp46, NKG2D) on CD56^+^ and CD16^+^ (NK cells) in chronic patients indicated an impairment of ADCC. Kaposi sarcoma-associated herpes virus (KSHV) evades NKp80-mediated cytotoxicity (40). However, the expression of these receptors in HIV controllers was similar to healthy donors and a higher level of plasma cytokine, which suggests that HIV controllers had better NK cytolytic activity than chronic patients. Also, NK cells with a high density of NKp46 are more efficiently lyse autologous, allogeneic, and xenogeneic target cells than NK cells that express low levels of NKp46 (41, 42). It was almost consistent with our result. In addition, HIV controllers expressed higher level of NKG2D compared to chronic patients. That means NKG2D may play an important role in HIV suppression through ADCC in HIV controllers. Recently, the activating NKG2D receptor was confirmed to influence ADCC responses (43). NKG2D functions as a triggering receptor involved in natural cytotoxicity mediated by normal NK cells against different tumors or normal target cells. Activating signals mediated by NKG2D vary from other receptors and were not susceptible to the negative regulatory signals by inhibitory receptors. Therefore, the individual activation of NKG2D is sufficient to stimulate the activation of NK cells and plays an essential role in it (44).Remarkably, the combined masking of NCR and NKG2D can reportedly lead to complete inhibition of NK-mediated lysis of all tumor or normal cells (45–48). The receptor down-regulation occurs during lytic viral replication and protects cells from NKG2D-mediated NK cytotoxicity (40). We found that NKp46 and NKG2D expression decreased in persons living with HIV and may explain why HIV-1 infected cells escape ADCC. Many previous studies demonstrated that ligands for the activating NK cell receptors NKG2D are induced during HIV-1 infection (45–48). Nirmin Alsahafi’s results suggested the involvement of NKG2D/NKG2D ligand interactions in the enhanced susceptibility of elite controller HIV-1-infected cells to ADCC responses (43). Higher expression of NKG2D in HIV controllers than chronic patients in our study concurs with Nirmin Alsahafi’s and Mair Thomas’ results. Some research showed that there is a dramatic expansion of CD56^-^/CD16^+^(CD56^-^) NK cells when HIV viral load is high (20, 49, 50), and it will decrease after antiviral therapy (14). Our result was partly consistent with them. NK cells functionality may decline, and immune surveillance of HIV-infected CD4 T-cells may be lost when cytotoxicity cells decrease and the unknown functional subset expands (13, 36). Several recent data has shown a consistent association between certain NK cell Killer Inhibitory Receptor (KIR) alleles and virus control, suggesting that NK cells may also have a functional role in controlling HIV replication (51, 52). However, in our study, inhibitory receptors have no changes in HIV controllers and chronic patients compared with healthy donors. The function of inhibitory receptors contributing to anti-HIV-1 ADCC remains open to debate.

Above all, our observation found HIV controllers performed higher level of activating receptors and plasma cytokines of IFN-γ, TNF-α and IL-12, which may potentially linked to the stronger killing function of NK cells. To up-regulate the expression of plasma cytokines and activating receptors of NK cells, especially NKG2D, may play a crucial part in killing and suppressing HIV. How to impair the down-regulation of ligands of NKG2D in viremic individuals may become a new thought for treatment or at least promise longevity. Thus, the further study will focus on CD56^+^CD16^-^NK cell subset in HIV controllers.

## Limitation

The number of HIV controllers among HIV infected patients is limited. The policy of Rapid ART made it even more difficult to find HIV controllers. The future holds promising challenges to discover new knowledges on the immune function within this group of patients. Given the importance of above factors, even though a limited number of HIV controllers were included in this study, our work shows its value supporting the advancement of researches in HIV controllers.

## Data Availability Statement

The original contributions presented in the study are included in the article/**Supplementary Material**. Further inquiries can be directed to the corresponding author.

## Ethics Statement

The Jiangsu Provincial Center for Disease Control and Prevention (JSCDC) ethics committee approved the study. All participants signed informed consents that reminded them of the right to decline participation or quit the study at any time before the face-to-face survey and blood collection. All experimental procedures followed the relevant guidelines and regulations approved by the Ethical Committee of JSCDC. The patients/participants provided their written informed consent to participate in this study.

## Author Contributions

Experimental design and manuscript preparation by G-FF and ZZ. Data analysis by Y-FC. ZZ, YZ, JL, H-YH, and X-QX performed the experiments. All authors contributed to the article and approved the submitted version.

## Conflict of Interest

The authors declare that the research was conducted in the absence of any commercial or financial relationships that could be construed as a potential conflict of interest.

## Publisher’s Note

All claims expressed in this article are solely those of the authors and do not necessarily represent those of their affiliated organizations, or those of the publisher, the editors and the reviewers. Any product that may be evaluated in this article, or claim that may be made by its manufacturer, is not guaranteed or endorsed by the publisher.
