# Supplementary figures and images for: Changes in NK Cell Subsets and Receptor Expressions in HIV-1 Infected Chronic Patients and HIV Controllers
Source: Front Immunol. 2021 Dec 16;12:792775. doi: 10.3389/fimmu.2021.792775 (PMC8716403; doi:10.3389/fimmu.2021.792775)

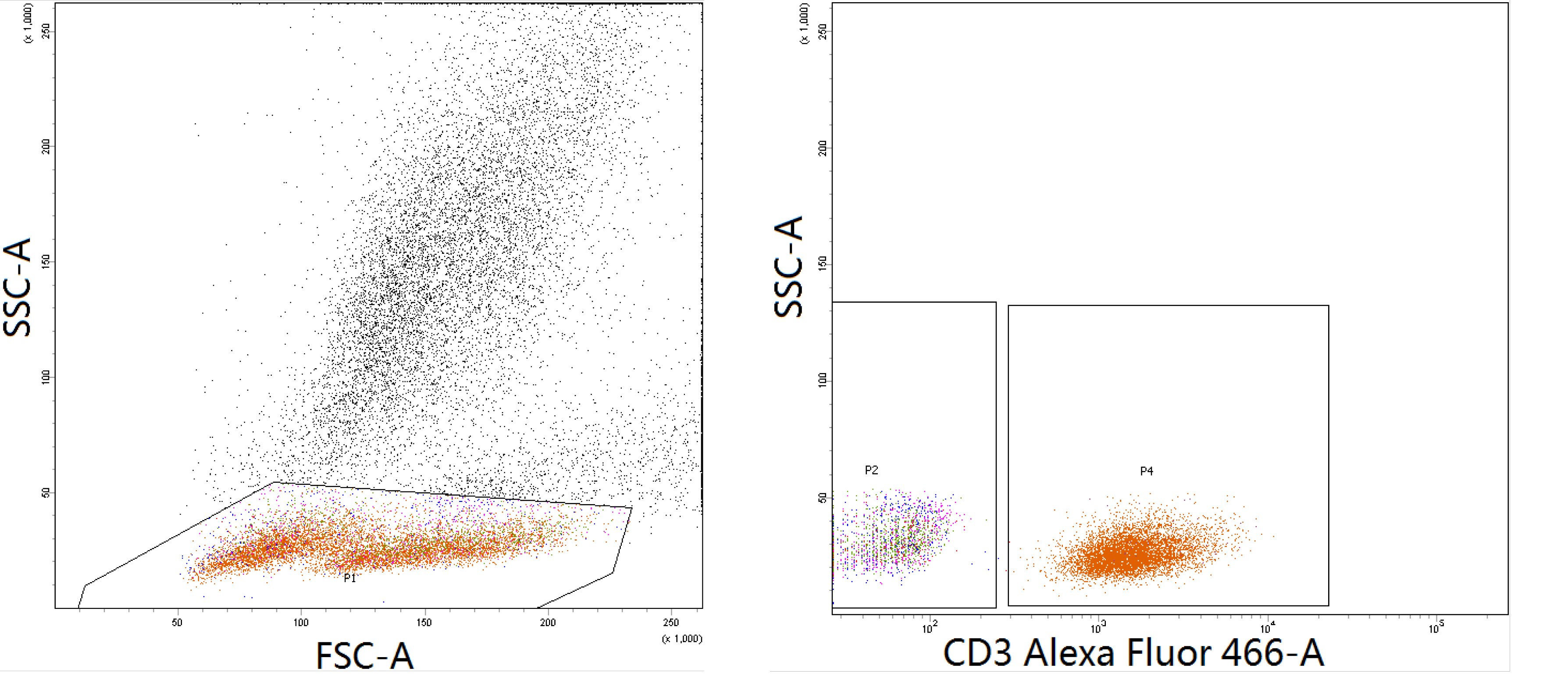

Supplement: Supplementary file 1 [file Image_1.tif]
